# Biochemical and physicochemical indicators of the quality of milk and meat obtained from cows with brucellosis

**DOI:** 10.14202/vetworld.2021.2118-2123

**Published:** 2021-08-17

**Authors:** Valeriy Alexandrovich Agoltsov, Stepan Yurievich Veselovsky, Olga Mikhailovna Popova, Tatiana Mikhailovna Giro, Nataliya Victorovna Solotova

**Affiliations:** Department of Veterinary Medicine and Biotechnology, Saratov State Agrarian University named after N.I. Vavilov, Saratov, Russia

**Keywords:** biochemical indicators, brucellosis, hydroxyproline, milk acidity, milk density, tryptophan

## Abstract

**Background and Aim:**

Brucellosis is a disease occurring worldwide. Although it is mainly a cattle disease, it is extremely dangerous for humans. Milk and meat can be contaminated with *Brucella*. The present study aims to examine the biochemical and physicochemical indicators of the quality of milk and meat obtained from cows positively reacting to brucellosis in comparison with healthy animals.

**Materials and Methods:**

Two groups of cattle meat samples (four muscles from different parts of the carcass) were obtained during slaughter at a specialized meat processing plant, and milk samples were examined from healthy animals (10 cows) and from cows positively responding to brucellosis (10 cows). For the milk samples, federal standards (GOST 32915-2014 and GOST 25179-2014) and an atomic absorption spectrometer “Kvant-Z ETA” were used. To evaluate the chemical composition of the meat, the “Clover” apparatus and a tissue grinder (SM-3) were used.

**Results:**

In the meat of cows that positively responded to brucellosis, compared with that of healthy animals, the amount of dry matter decreased by 1.2 times, amino ammonia nitrogen by 1.01 times, proteins by 1.2 times, fat by 1.28 times, volatile fatty acids by 1.09 times, tryptophan by 1.25 times, oxyproline by 1.14 times, and protein quality indicator by 1.21 times.

**Conclusion:**

Despite the severity of brucellosis in cattle, the biochemical and physicochemical indicators of the quality of milk and meat obtained from the healthy and contaminated animals vary, although insignificantly.

## Introduction

In recent years, the number of animals with brucellosis tends to increase globally [1]. Many cases of this disease are noted in Kazakhstan, Kyrgyzstan, Uzbekistan, and Russia [2]. However, the greatest distribution of brucellosis is in Africa, Central and South America, in some countries of Asia, and Europe [3]. The peculiarity of this disease is that it is extremely dangerous for humans [4]. The epizootic process is boosted by the transmission of brucellosis through milk [5] and meat contaminated with *Brucella* [6]. Therefore, the slaughtering and butchering of sick animals are possible only at specialized meat processing plants, observing the rules for working with animals with brucellosis [7]. Because of the complexity of the epizootic situation and the inability to completely eliminate the disease, even with the use of various anti-brucellosis vaccines [8], many carcasses from animals that positively responded to serological reactions are annually delivered to meat processing plants. Boiled sausages are mainly made from the meat of such animals [9,10]. The condition of carcasses obtained from animals with brucellosis also raises many questions on their fatness and the nutritional value of their meat [11]. Therefore, the system of complex control of animal brucellosis and the ways of its spread should include the fight against brucellosis in meat processing plants [12,13]. The nutritional value of meat obtained from animals with brucellosis requires a large-scale biochemical study [14].

There is no information on the biochemical changes in meat obtained from cattle positively responding to brucellosis. Thus, the study of the main biochemical parameters of the quality of meat obtained from cattle with brucellosis is extremely relevant [15].

Animal milk is also of great importance in human nutrition. Many people enjoy using this product in their diet. Milk is rich in fats, proteins, vitamins, and macro- and micro-elements that people need for their normal life [16,17]. However, various infectious diseases, including brucellosis, can also be transmitted through milk, dairy products, and meat [18]. Therefore, milk should not only be considered a valuable food for people but also as a potential source of human infection with brucellosis [19]. There is no information on the biochemical changes in milk obtained from cows that positively react to brucellosis in serological reactions. Thus, the study of the main biochemical parameters of the quality of meat obtained from cattle with brucellosis is also relevant [20].

The present study aims to examine the biochemical and physicochemical indicators of the quality of milk and meat obtained from cows positively reacting to brucellosis in comparison with healthy animals.

## Materials and Methods

### Ethical approval

Ethical approval for this study was obtained from the Animal Ethics Committee of the Faculty of Veterinary Medicine and Biotechnology, Saratov State Agrarian University, Saratov, Russia. Ethical clearance certificate number 207 protocol 8, dated July 20, 2020. Experimental research, maintenance, care, and euthanasia were carried out in accordance with the requirements of the “European Convention for the Protection of Vertebrate Animals used for Experimental and Other Scientific Purposes” (1986).

### Study period and location

The study was conducted in August 2020 and September 2020. The slaughter of bovine cattle sick with brucellosis was carried out at a specialized slaughterhouse LLP “Tandem” in the Aktobe region of the Republic of Kazakhstan, the farm “Syrym.”

### Animals

The study included 20 cattle: 10 cows that positively reacted to brucellosis when examining the blood of all cattle of the farm (experimental group) and 10 clinically healthy animals (control group). These 20 cows’ milk was studied, and their meat was then examined after slaughter.

### Sampling

Two groups of cow meat samples obtained during slaughter at a specialized meat processing plant were examined:

1.Meat obtained from healthy animals (10 cows) and
2.Meat obtained from animals that positively responded to brucellosis (10 cows).

To evaluate the chemical composition of the meat, a part of the muscle tissue was obtained from four muscles from different parts of the carcass (brachiocephalic, triceps muscles, and lumbar part of the longissimus dorsi and biceps femoris) and combined into a general sample. The muscles were freed from fascia and fat. The selected average samples were subjected to chemical research according to generally accepted methods [21].

The samples obtained for analysis were passed through a tissue grinder (SM-3) 3 times and thoroughly mixed. The minced meat prepared in this way was used for weighing. The sampling of meat from animals of both groups for biochemical analysis was conducted at the meat processing plant.

### Biochemical assays

The fat mass fraction and acidity of milk were evaluated in accordance with GOST 32915-2014 Milk and Dairy Products (The Russian Federal Standard). The protein mass fraction was evaluated according to GOST 25179-2014 Milk and Dairy Products (The Russian Federal Standard).

The amount of macro- and micro-elements was determined using an atomic absorption spectrometer, “Kvant-Z ETA” (Cortec Ltd., Moscow, Russia), according to the standard method [22]. The density, acidity, fat, and protein were evaluated using the “Clover” apparatus (Infraspec Ltd., Krasnoobsk, Russia). The meat was studied 24 h after the slaughter of the animals and stored in a refrigerator at a temperature of 2°C-4°C.

In the course of the experiments, the following biochemical parameters of meat quality were investigated: Dry matter, AAA proteins, fat, protein, volatile fatty acids (VFAs), moisture, ash, tryptophan, hydroxyproline, and protein quality indicator (PQI). These parameters were obtained from healthy cattle and those that positively reacted to brucellosis [23].

### Statistical analysis

Statistical data processing was conducted using the “Analysis Package” of the MS Excel spreadsheet processor [24]. The reproducibility of the results of the biochemical parameters was established on the basis of the Cochran test, and using the Fisher criterion, it was shown that the chosen mathematical model corresponds to the experimental data with a 95% level of reliability. The analysis of variance of the data was obtained, and the significance level was set at p≤0.05.

## Results

**Figure-1:**
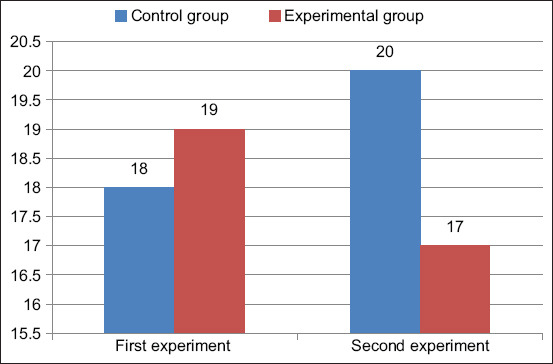
Cow milk acidity (°T).

The milk from 10 cows (experimental group) that positively responded to brucellosis was sampled twice with an interval of 4 days. The control was the milk from 10 healthy cows (control group). The acidity of cow milk is shown in Figure-1. The experiment was conducted twice. The acidity of the milk from healthy cows was 18°T (Therner degrees) in the first experiment and 20°T in the second experiment, which corresponds to the physiological value of the acidity of fresh milk. The acidity of the milk from cows that positively responded to brucellosis was 19°T in the first experiment and 17°T in the second experiment, which also corresponds to the acidity of uncooled milk obtained from cows after 2 h or more.

**Figure-2:**
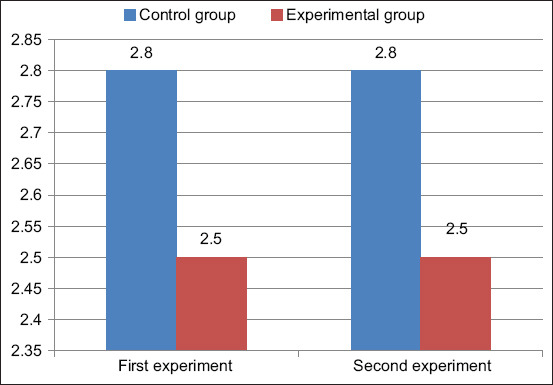
Cow milk fat, %.

The fat content of cows’ milk is shown in Figure-2. The fat content of the milk from cows of the control group was 2.8% in both the first and second experiments, which corresponds to the classic fat content of milk from cows. The fat content of the milk from the experimental group was 2.5% in the first and second experiments, which corresponds to low-fat milk. Thus, the milk from cows that positively reacted to brucellosis was less fatty than that from healthy cows.

**Figure-3:**
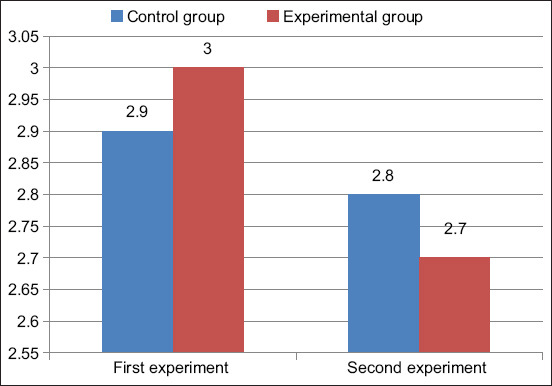
Cow milk protein, %.

The protein content in cow’s milk is shown in Figure-3. The amount of protein in the milk of the control group was 2.9% in the first experiment and 3.0% in the second experiment. In the milk of cows that positively reacted to brucellosis, the protein content was 2.8% in the first experiment and 2.7% in the second experiment, which is slightly below the physiological norm for milk protein.

**Figure-4:**
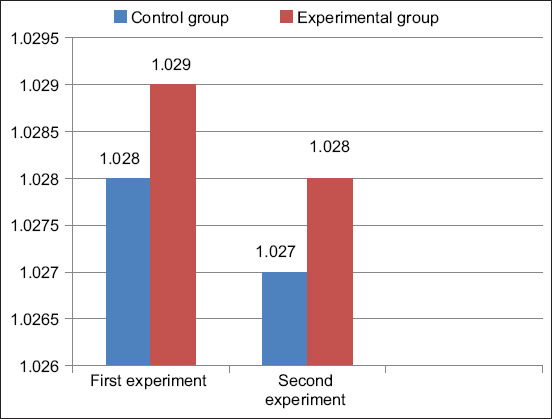
Cow milk density, g/cm^3^.

The milk density of the control group (Figure-4) was 1.028 g/cm^3^ in the first experiment and 1.029 g/cm^3^ in the second experiment, while the milk density of the experimental group was 1.027 g/cm^3^ in the first experiment and 1.028 g/cm^3^ in the second experiment. The milk density of the control and experimental groups differs from each other only by insignificant numbers, and in both groups, the values do not exceed the physiological norm (1.027 g/cm^3^).

The data from the study of the biochemical indicators of the quality of cow meat of both groups are presented in Table-1. The dry matter in the meat of the control animals comprised 25.2%. The same indicator in the meat of animals of the experimental group on average was lower; the difference between the indicators of the two groups was 4.1%, which is 1.2 times higher than in the meat of the experimental group. On average, the amino ammonia nitrogen was 70.2% in the meat of the animals in the control group and 69.4% in the meat of the animals in the experimental group, which is 1.01 times lower than in the meat of the control group.

**Table-1:** Biochemical indicators of the quality of beef obtained from healthy cows and from those responding positively to brucellosis (average indicators).

Biochemical indicators	Group (n=10)	

	Control group	Experimental group
Dry matter, %	25.2±0.1	21.1±0.48
Amino ammonia nitrogen, %	70.2±0.26	69.4±0.2
Fat, %	14.1±0.24	11.04±0.16
Protein, %	19.2±0.15	15.9±0.22
VFA, mg	2.3±0.23	2.1±0.15
Moisture, %	72.4±1.15	72.7±1.5
Ash, %	0.9±0.18	1.1±0.52

p=95%, VFA=Volatile fatty acids

The fat content in the meat of the cows in the control group was 14.1%. Its value in the meat of animals in the experimental group was significantly lower and amounted to 11.04%, which is 1.28 times lower than the meat of the control group. This indicator is lower in the second group, since the carcasses of animals that positively responded to brucellosis were less well-fed (thin) compared with the carcasses of the healthy animals. The protein content was also significantly higher in the meat of the control group (19.2%) compared with the meat of the experimental group, where it was 15.9%, which is 1.2 times higher than the indicators of the experimental group. All these, in our opinion, are also associated with the low fatness of animals with brucellosis. In the process of illness, the animal experiences pain, eats poorly, and is exhausted.

The content of VFAs in the meat of the animals in the control group was 2.3 mg. In the meat of the cows of the experimental group it was reduced, in comparison with the first value, by 1.09 times. We also believe that a slight decrease in the content of VFAs in the meat of animals in the second group is due to the lack of these acids in the body of animals that positively responded to brucellosis.

The moisture content in the meat of the cows in the control group was 72.4%. This indicator in the experimental group was slightly lower in comparison with the first figure and amounted to 72.2%, which is almost equal. The amount of ash in the meat of the animals in the control group was 0.9%. This indicator in the meat of the animals in the experimental group was 1.22 times higher than in the control one and amounted to 1.1%.

The tryptophan content in the meat of animals in the control group was 355.8 mg%, which corresponded to the physiological value for this type of meat. Its value in the meat of animals in the experimental group was reduced and amounted to 284.3 mg%, which is 1.25 times lower than in the meat of the control group of animals. All these data indicate that animals that positively reacted to brucellosis have poor appetite and do not consume enough feed, leading to the lack of tryptophan in the body.

**Figure-5:**
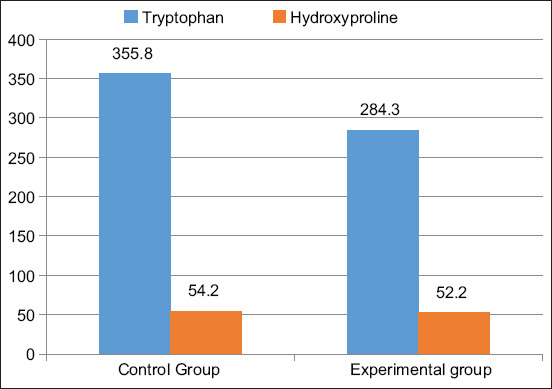
Tryptophan and hydroxyproline content in meat, mg%.

The level of hydroxyproline in the meat of the animals in the control group was 54.2 mg%. The content of hydroxyproline in the meat of the cows in the experimental group was lower and amounted to 52.2 mg%, which is 1.14 times lower in relation to the meat of the control group. The level of hydroxyproline in the meat of the cows in the experimental group corresponded to the lower limit of the physiological norm (Figure-5).

**Figure-6:**
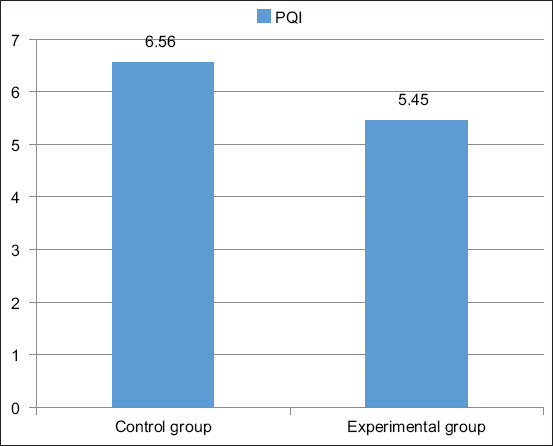
Protein quality index of meat, %.

The potential biological value of protein can be judged by the value of the PQI of meat, which is the ratio of the amount of tryptophan to hydroxyproline. This indicator allows judging the ratio of muscle and connective tissue proteins. The PQI for beef meat is normally 6.4. In the meat of the control group, this figure was 6.6, which is close to the norm. In the meat of the experimental group, this indicator was 5.45, which is slightly lower than the norm and, accordingly, 1.15 lower than the meat of the control group. The reduced PQI in the meat of the experimental group is associated with a decrease in feed consumption and metabolic disorders in animals with brucellosis. The PQI is presented in Figure-6.

## Discussion

It is well known that, under brucellosis, metabolic disorders are noted. They are reflected in the quantitative and qualitative indicators of milk and meat in cow [25]. The present study confirms those changes as well. Agreeably, other researches confirm such data through other diseases; for example, in cow mycotoxicosis caused by associated T-2 and aspergillotoxicosis, there are deep complex violations of the mineral balance of the body. They are manifested in a decrease in the content of the main macro- and micro-elements not only in the body but also in cow’s milk. Studying the biochemical parameters of meat obtained from an animal with associated T-2 and aspergillotoxicosis of cattle affected by other infectious diseases, researchers found significant deviations in physical, chemical, and biochemical parameters [26,27]. However, the present study of the corresponding parameters from cows that positively reacted to brucellosis did not show significant deviations.

Previous study compared the presence of moisture and protein in ostrich meat with meat obtained from cows that positively responded to brucellosis and concluded that the prior contained more protein (21.4%) and moisture (76.1) than did the latter (protein: 15.9% and moisture: 72.7%) [28].

The moisture content of quails and turkeys grown in the Krasnodar region of the Russian Federation was 67.93% in quails, which is slightly lower than in the meat of cows that positively responded to brucellosis (72.7%), and 75.92% in turkeys, which is slightly higher than in the meat of cows with brucellosis. The protein content in quail meat was 21.92%, which is significantly higher than in the meat of cows that positively responded to brucellosis (15.9%), and in turkey meat, it was 13.76%, which is lower compared with the meat of cows with brucellosis [29].

Our findings correspond with the research results of other scientists in the fact that, in the milk of cows suffering from infectious diseases, such as brucellosis and mycotoxicosis, the amount of macro- and micro-elements decreases [30]. The researchers state that fodder mycotoxicosis against the background of impaired mineral metabolism contributes to a significant change in the balance of the biochemical indicators of meat quality, manifested by a decrease in the level of dry matter by 1.18 times (by 4.0%), fat by 1.38 times (by 1.0%), protein by 1.34 times (by 5.0%), ash by 1.68 times (by 0.49%), tryptophan by 1.26 times (by 74.3%), and PQI-BCP by 1.42 times (by 2.03 units) and an increase in AAA meat by 1.09 times (by 7.0%), VFAs by 1.47 times (by 1.04 mg), moisture by 1.06 times (by 4.7 %), hydroxyproline by 1.12 times (by 6.2%), and pH by 1.19 times (by 1.1 units) [31]. All the findings correlate with our results and state that infectious diseases change the biochemical and physicochemical indicators of the quality of milk and meat obtained from cows that positively reacted to brucellosis.

## Conclusion

In the meat of cows that positively responded to brucellosis, compared with that of healthy animals, because of metabolic disorders, the amount of dry matter decreases, while the amount ash content increases. The moisture content of the two groups of animal meat was practically equal. In the milk of the cows with brucellosis, compared with the milk obtained from healthy cows, the acidity was higher. The amount of fat was 2.5 %, which corresponds to low-fat milk. The protein content was 2.8% in the first experiment and 2.7% in the second experiment, which is slightly below the physiological norm for milk protein. The milk density of the experimental groups was different by insignificant numbers.

Therefore, despite the fact that various infectious diseases were examined and that apart from cows, different animals were observed, the majority of studies indicate changes in the biochemical parameters of meat and milk obtained from cows with brucellosis. However, the present study displays the change as not so significant compared with meat and milk obtained from healthy animals. Therefore, it is possible to determine the most rational ways of processing disinfected milk for on-farm needs.

## Authors’ Contributions

TMG and OMP: Designed the experiment. SYV and VAA: Conducted the statistical analysis, as well as conducting the experimental measurement. NVS: Contributed in writing the article and reviewing it thoroughly before submission. All authors read and approved the final manuscript.
